# Full-length transcriptome sequencing provides insights into alternative splicing under cold stress in peanut

**DOI:** 10.3389/fpls.2024.1362277

**Published:** 2024-03-07

**Authors:** Xin Wang, Yue Liu, Lei Ouyang, Ruonan Yao, Tingting Yu, Liying Yan, Yuning Chen, Dongxin Huai, Xiaojing Zhou, Zhihui Wang, Yanping Kang, Qianqian Wang, Huifang Jiang, Yong Lei, Boshou Liao

**Affiliations:** ^1^Key Laboratory of Biology and Genetic Improvement of Oil Crops, Ministry of Agriculture and Rural Affairs, Oil Crops Research Institute of the Chinese Academy of Agricultural Sciences, Wuhan, China; ^2^State Key Laboratory of Biocatalysis and Enzyme Engineering, School of Life Sciences, Hubei University, Wuhan, China

**Keywords:** peanut, cold stress, full-length transcriptome, alternative splicing, splicing factor

## Abstract

**Introduction:**

Peanut (*Arachis hypogaea* L.), also called groundnut is an important oil and cash crop grown widely in the world. The annual global production of groundnuts has increased to approximately 50 million tons, which provides a rich source of vegetable oils and proteins for humans. Low temperature (non-freezing) is one of the major factors restricting peanut growth, yield, and geographic distribution. Since the complexity of cold-resistance trait, the molecular mechanism of cold tolerance and related gene networks were largely unknown in peanut.

**Methods:**

In this study, comparative transcriptomic analysis of two peanut cultivars (SLH vs. ZH12) with differential cold tolerance under low temperature (10°C) was performed using Oxford Nanopore Technology (ONT) platform.

**Results and discussion:**

As a result, we identified 8,949 novel gene loci and 95,291 new/novel isoforms compared with the reference database. More differentially expressed genes (DEGs) were discovered in cold-sensitive cultivar (ZH12) than cold-tolerant cultivar (SLH), while more alternative splicing events were found in SLH compared to ZH12. Gene Ontology (GO) analyses of the common DEGs showed that the “response to stress”, “chloroplast part”, and “transcription factor activity” were the most enriched GO terms, indicating that photosynthesis process and transcription factors play crucial roles in cold stress response in peanut. We also detected a total of 708 differential alternative splicing genes (DASGs) under cold stress compared to normal condition. Intron retention (IR) and exon skipping (ES) were the most prevalent alternative splicing (AS) events. In total, 4,993 transcription factors and 292 splicing factors were detected, many of them had differential expression levels and/or underwent AS events in response to cold stress. Overexpression of two candidate genes (encoding trehalose-6-phosphatephosphatases, AhTPPs) in yeast improves cold tolerance. This study not only provides valuable resources for the study of cold resistance in peanut but also lay a foundation for genetic modification of cold regulators to enhance stress tolerance in crops

## 1 Introduction

Cultivated peanut (*Arachis hypogaea* L., also called groundnut) is an important grain legume grown in more than 100 countries worldwide. Peanut seeds contain a rich source of beneficial nutrients for human health, including vegetable oil, protein, sugar, and minerals. Although the global planting area of peanut has reached 31.60 million hectares, with an annual production of approximately 50 million tons, it still cannot meet the increasing demands of consumers due to the growing population and adjustment of agricultural structure. Since peanut presumably originates from tropical regions of South America (Naito et al., 2008), it requires relatively warm temperature throughout overall developmental stages (Bell et al., 1994). In northeast of China, extreme low temperature caused by global climate change occurs frequently in the early spring sowing seasons, leading to a serious decline in peanut yields (Kakani et al., 2002; Shen et al., 2019). Thus, cold stress is considered as one of major limiting factors that adversely affect peanut growth and restrict planting areas. Despite being a thermophilic crop, the cold tolerance of some peanut cultivars was found to be significantly different (Upadhyaya et al., 2009). So far, only few germplasms and cultivars with strong cold tolerance have been discovered or developed (Zhang et al., 2023). Therefore, it is crucial to investigate the mechanism of peanut cold tolerance and cultivate new cold-resistant varieties to enhance its production capacities (Bresolin et al., 2008).

Low temperature inhibits photosynthetic activity and reduces water uptake. During cold stress, reactive oxygen species (ROS) was excessively generated in chloroplasts, mitochondria, and peroxisomes, with detrimental damages on plasma membrane (Zhang et al., 2019). However, plants evolve a series of strategies to defense against low temperature, including physiological and biochemical changes and sophisticated molecular regulatory mechanisms (Ding et al., 2019). The most extensively studied cold stress regulatory pathway was the CBF/DREB1-COR signaling module. The C-repeat binding factors/dehydration-responsive element-binding proteins (CBFs/DREBs) belong to AP2/ERF (APETALA 2/ethylene response factor) transcription factor family members and was involved in cold response (Kim et al., 2015). In general, the expression levels of CBFs were sharply induced by low temperature; then, CBFs activated the transcriptional expression of downstream cold-responsive (COR) genes via binding to their promoter cis-acting elements (Tang et al., 2020).

Whole genome re-sequencing (WGRS) has accelerated the process of mapping and cloning of genes that control important traits in many crops. Recently, a major quantitative trait locus (*qRGRB09*) governing peanut seed germination at low temperature was finely mapped in a 216.26-kb region on chromosome B09, wherein 15 candidate genes were discovered (Zhang et al., 2023). In addition, transcriptomics based on second-generation sequencing (SGS) was proved to be a highly effective approach to reveal COR genes (Sinha et al., 2015; Wu et al., 2016; Jin et al., 2017; Li et al., 2019). Transcriptomic analysis of peanut cultivars with contrasting cold tolerance identified several COR genes and provided insights into cold signal transduction pathways (Zhang et al., 2020). However, the relatively short reads produced by SGS are not conducive to bioinformatic analysis, thus reducing the accuracy of sequence assembly. With the development of technology, third-generation sequencing technologies are available, including Pacific Biosciences (PacBio) (Rhoads and Au, 2015) and Oxford Nanopore Technologies (ONT) (Michael et al., 2018; Jain et al., 2018). Compared with PacBio platform, ONT is more cost effective for the characterization of full-length transcriptome and quantification of transcript expression in plants (Cui et al., 2020).

Alternative splicing (AS) is an important regulatory process of gene expression, in which multiple mRNA isoforms are produced from an RNA precursor (pre-mRNA) through different combinations of exons and introns (Blencowe, 2006). This process can generate various kinds of novel transcripts and protein variants (Black, 2000). Previous studies have reported that AS is participated in plant stress responses, including cold, salt, and other abiotic stresses (Laloum et al., 2018). Proper alternative splicing of pre-mRNAs controlled by the spliceosome components, including the small nuclear ribonucleoproteins (SnRNPs) and arginine/serine-rich splicing factors (SPs), is critical for plant adaptation to low temperatures (Jabre et al., 2021; Zhao et al., 2021; Zhang et al., 2022). It has been reported that 27% of chilling-response genes undergo AS events in *Arabidopsis* (Calixto et al., 2018). The long reads obtained by ONT sequencing could accurately characterize AS transcript isoforms and offer a novel transcriptomic reference basis for gene functional assays.

The cold tolerance of peanut is a highly complex trait, which is genetically controlled by multiple quantitative trait loci and/or genes (Zhang et al., 2019, 2023). In the current study, to get new insight into the molecular mechanisms underlining cold tolerance of peanut, we analyzed transcript expression changes and differential alternative splicing (DAS) genes in the two cultivars with contrasting cold resistance (SLH and ZH12) by using ONT sequencing platform. This study facilitates better understanding of cold-responsive pathways and the functions of AS in peanut and thus be useful for developing novel cultivars with strong cold tolerance.

## 2 Materials and methods

### 2.1 Plant materials and cold treatment

Two *Arachis hypogaea* cultivars (Silihong and Zhonghua 12) with differential cold tolerance were used in this study (Wang et al., 2021; Zhang, et al., 2021). Peanut seeds were soaked in distilled water for 6 h, potted in soil, and then placed into a growth chamber under a 16 h/8 h (light/dark) cycle at 28°C. Two-week-old seedlings were subjected to cold stress (10°C). The third unfold leaves from seedlings were collected after 0 h, 3 h, 12 h, and 24 h. All samples with three biological replicates were quickly frozen in liquid nitrogen and stored at −80°C until use.

### 2.2 ONT transcriptome sequencing

Total RNA was extracted from peanut leaves treated with and without cold stress treatment. RNA integrity and concentrations were detected by the Bioanalyzer 2100 system (Agilent Technologies, CA, United States). One microgram of total RNA for each sample was prepared for cDNA library according to the protocol provided by Oxford Nanopore Technologies (ONT). The cDNA libraries were added to flowcells (FLO-MIN109) and run on an ONT sequencing platform (PromethION 24, P24) at Biomarker Technology Company (Beijing, China). The raw reads derived from 24 samples have been deposited in the NCBI SRA database with accession number of PRJNA1032881. Low quality (length <500 bp, Q-score <7) of raw reads and ribosomal RNA sequences were first filtered. Then, the full-length transcripts were determined by searching for clean reads that have ONT library primers at both ends. Full-length transcripts were obtained by mapping to reference genome (cv. Tifrunner, https://data.legumeinfo.org/Arachis/hypogaea/genomes/Tifrunner.gnm2.J5K5, accessed on April 4, 2023) with mimimap2 software. Mapped reads were further collapsed by cDNA_Cupcake package with minimum coverage of 85% and minimum identity of 90%. The duplicated transcripts were removed.

### 2.3 Transcriptome annotation and functional enrichment analysis

The obtained full-length transcripts were annotated with the following databases: NR (NCBI non-reductant protein sequences), Swiss-port database, Pfam (protein family), Gene Ontology (GO), and KEGG (Kyoto Encyclopedia of Genes and Genomes). GO enrichment analysis was performed by the GOseq R packages (Young et al., 2010). KEGG enrichment analysis was implemented by KOBAS software (Mao et al., 2005).

### 2.4 Screening of differentially expressed genes and DAS events

Transcripts were validated against available reference transcriptome annotations (https://www.peanutbase.org/download/) with gffcompare. Expression levels were estimated by reads per gene/transcript per 10,000 reads mapped (CPM). DEGs were screened using the DESeq2 R package (1.6.3) with the criteria of |log2FC (fold change)| > 1 and an adjusted *p*-value < 0.01 (Love et al., 2014). Alternative splicing (AS) events were analyzed by the AStalavista tool (Foissac and Sammeth, 2007), and DAS events were identified by PSI-sigma tools (Lin and Krainer, 2019). Transcription factors were detected from the transcriptome data using iTAK software (Zheng et al., 2016).

### 2.5 qRT-PCR and RT-PCR

Approximately 1 µg of total RNA (DNase I-treated) was used for first-strand cDNA synthesis for each sample with MMLV reverse transcriptase kit (Thermofisher Scientific, United States). RT-PCR was used for analysis of the alternatively spliced isoforms, and real-time quantitative RT-PCR (qRT-PCR) was performed to analyze the expression level of DEGs. qRT-PCR reactions were carried out on the Bio-Rad CFX96 RT-PCR Detection system (Bio-Rad, Hercules, CA, United States) using Hieff qPCR SYBR Green Master Mix. The relative gene expression levels were quantified by 2^—△△Ct^ method (Livak and Schmittgen, 2001), using *A. hypogaea* Actin gene (accession number *Aradu.W2Y55*) as an internal reference gene for normalization. The expression level of 0 h in SLH was used as a control, whose value was set to 1. All primers used are listed in **Supplementary Table S1**.

### 2.6 Verification of candidate genes function by yeast expression system

The coding sequences of *AhTPP* genes (*Arahy.FIK9JS*, *Arahy.0FY2NM*) were amplified by PCR using peanut leaf cDNA as template with primers listed in **Supplementary Table S1**. The PCR products were purified and cloned into yeast expression vector (pYES2). The resulting *pYES2-AhTPPs* plasmids were transformed into yeast cells (INVSc1) individually. For cold tolerance assays, the transgenic yeast cultures harboring empty vector pYES2 (control) or *pYES-TPPs* were first subjected to −20°C for different times (0 h, 24 h, 48 h, 72 h, and 96 h) and serially diluted with SG-U liquid medium (synthetic complete without uracil medium + 2% galactose + 2% agar) to different concentrations (10^0^, 10^−1^, 10^−2^, and 10^−3^); then, 2 μL of each culture was spotted onto SG-U medium plates. The plates were photographed after incubating at 28°C for 72 h.

## 3 Results

### 3.1 Novel genes and isoforms identified by ONT sequencing

Peanut cultivar Silihong (SLH) is popularly planted in northeast China. Zhonghua 12 (ZH12) is a representative variety released by Oil Crops Research Institute, Chinese Academy of Agricultural Sciences. In a previous study, SLH was shown to have better cold tolerance than ZH12 (Zhang et al., 2021). First, both the two cultivars showed similar seed germination rates (>90%) under normal condition. The germination rate was severely down to approximately 50% in ZH12 (cold susceptible) after 72 h cold treatment, but it was not affected in SLH (cold tolerant). Second, the levels of reactive oxygen species (H_2_O_2_ and O_2_^−^) and contents of malondialdehyde (MDA, an important indicator for cell membrane damage caused by oxidative stress) were significantly lower in the seedlings of SLH than those in ZH12 under cold stress. Thus, the differential performance and physiological response to low temperature indicated that SLH was relatively more cold-resistant than ZH12 (Wang et al., 2021).

The long-read ONT sequencing platform could theoretically obtain the full-length transcript information without requiring fragmented RNA. To investigate the molecular basis of peanut in response to cold response, we use ONT sequencing to analyze the transcriptome of SLH and ZH12 after cold treatment for different time (0 h, 3 h, 12 h, and 24 h). As a result, a total of 168 million clean reads derived from the 24 samples (three biological replicates per time points) were obtained, with an average length of 1,040–1,393 nucleotides per each sample (**Supplementary Table S2**). The number of full-length transcripts varied from 4.5 to 8.6 million per library, accounting for more than 84% of clean reads (**Supplementary Table S3**), indicating that the data were good enough for following bioinformatic analysis.

Full-length transcriptome based on ONT sequencing could facilitate to optimize the structure of transcripts by alignment of long-read transcripts with the reference genome and/or transcriptome of peanut cv. Tifrunner (Bertioli et al., 2019). When mapped to the genome sequences, a total of 38,511 genes were discovered in our transcriptome data, including 8,949 novel gene loci (**Supplementary Table S4**). Some novel genes with high expression levels were found to be associated with stress response. For example, ONT.16282 was annotated as a homolog of abscisic stress-ripening protein 2 (ASR2), which confers tolerance to drought in plants (Srivastava et al., 2022). In addition, 125,159 redundant-removed consensus transcript sequences were obtained through minimap2 software. Among of them, only 29,958 transcripts (23.93% of total transcripts) were known isoforms, while 81,364 (65.0%) were new isoforms mapped to the known genes, and 13,837 (11.05%) were considered as novel isoforms that mapped to unannotated genomic regions (**Supplementary Table S5**). The results indicated that ONT sequencing could be used as an efficient and robust tool to provide significant additional annotations to the available reference genome.

### 3.2 Identification of DEGs in response to cold stress

**Figure 1:**
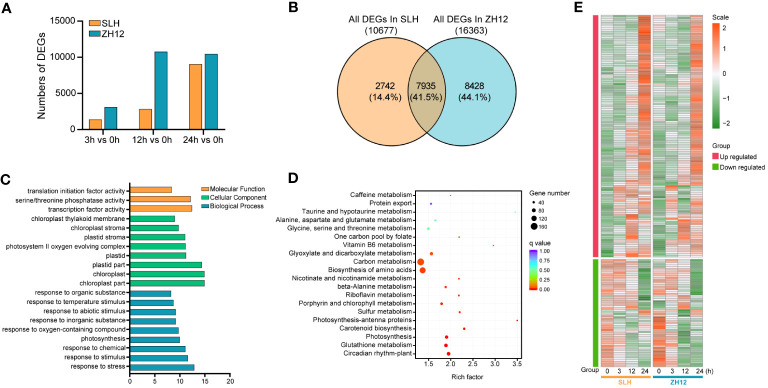
Analysis of differentially expressed genes in the two peanut cultivars. **(A)** The counts of differentially expressed genes (DEGs) between cold stress (3 h, 12 h, and 24 h) and normal condition (0 h). **(B)** Venn diagram of all DEGs in SLH and ZH12. The percentage indicates the number of DEGs in each section accounted for the total number of DEGs in the two cultivars. Gene Ontology **(C)** and KEGG **(D)** enrichment of the 7,935 common DEGs in the two cultivars. **(E)** Heatmap depicting expression pattern of DEGs in the most enriched GO term “response to stress”.

**Figure 2:**
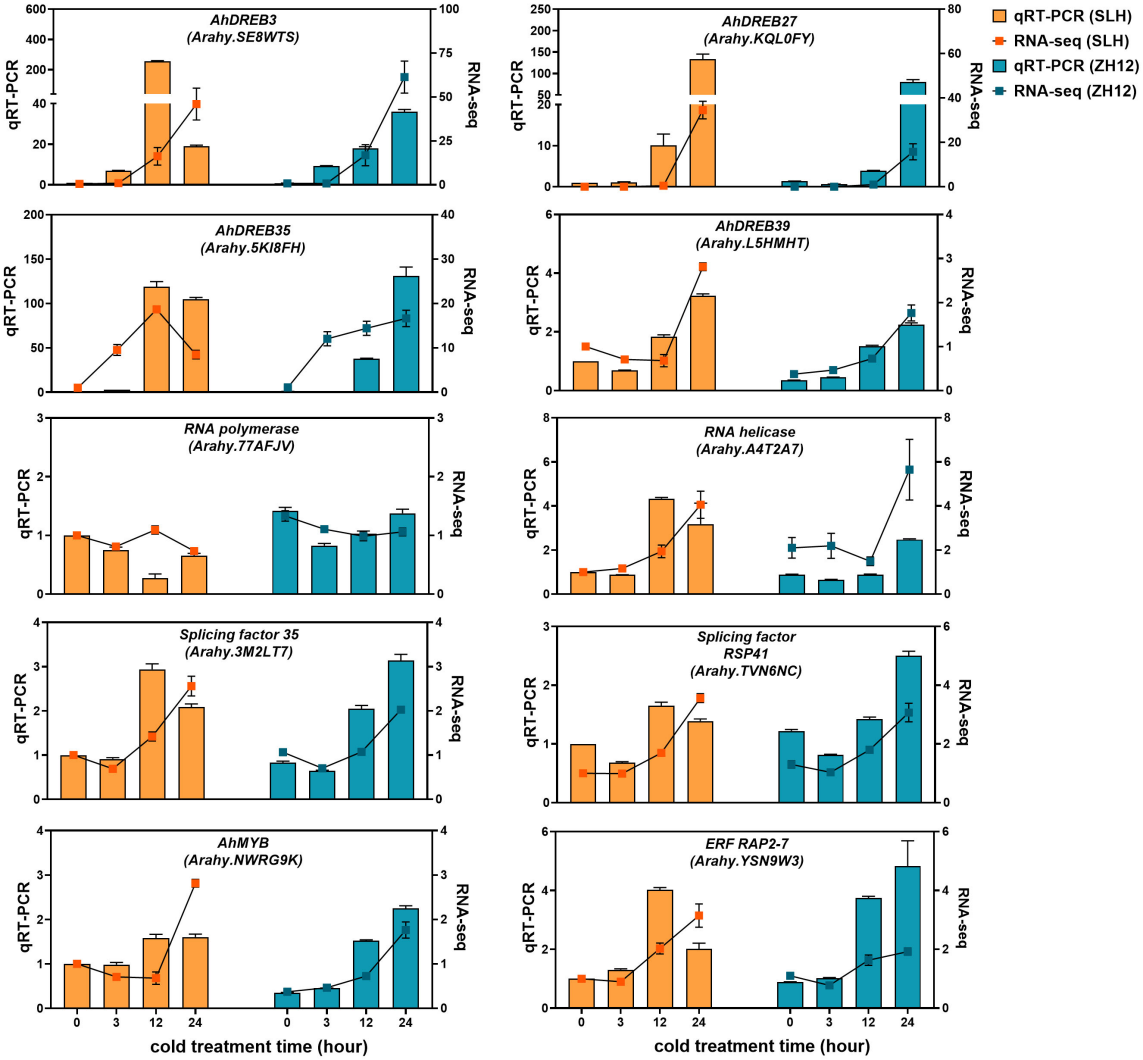
qRT-PCR validation of differentially expressed genes in the two peanut cultivars. The relative transcript levels among different samples were quantified by the 2^−ΔΔCt^ method, with *A. hypogaea* Actin gene as the reference gene for normalization. No treatment control (0 h) in SLH was normalized as “1”. Data are means ± SE of three biological replicates.

The cold-responsive genes were identified by screening DEGs between cold-treated samples (3 h, 12 h, and 24 h) and normal control (0 h). In both cultivars, the number of DEGs in 12 h or 24 h was greater than that in 3 h (**Figure 1A**), suggesting that prolonged duration of cold treatment led to more profound alternations of transcriptome profiles in peanut. There was a total of 10,677 and 16,363 DEGs in the three comparison sets (24 h vs. 0 h, 12 h vs. 0 h, 3 h vs. 0 h) of SLH and ZH12 respectively (**Supplementary Table S6**), and 7,935 common DEGs were shared between the two cultivars (**Figure 1B**). We performed GO analysis of these common DEGs to explore their possible functional processes. The most enriched GO terms associated with “biological process” categories was “response to stress” (GO:0006950) (**Figure 1C**; **Supplementary Table S7**). Most stress-associated genes were significantly upregulated by low temperature in this GO term (**Figure 1E**; **Supplementary Table S8**), including glutathione S-transferases (*Arahy.F8LB2J*, *Arahy.67E0CQ*), suggesting their vital roles in peanut cold resistance. The top frequent GO terms belonging to “cellular component” and “molecular function” categories were “chloroplast part” (GO:0044434) and “transcription factor activity” (GO:0003700), respectively, indicating that photosynthesis process and transcription factors play vital roles in cold stress response in peanut. KEGG enrichment analysis showed that “circadian rhythm-plant” (ko04712) and “glutathione metabolism” (ko00480) were most abundantly enriched (**Figure 1D**). Moreover, 2,742 DEGs were found specifically in SLH. “Transketolase activity” (GO:0004802) was the most enriched GO term, and biosynthesis of amino acids (ko01230) was the top enriched pathway (**Supplementary Figure S1**). This result suggested that the pentose phosphate pathway and protein metabolisms were more associated with cold response in resistant peanut variety (SLH). To verify the reliability of the expression profiles of DEGs from ONT sequencing data, 10 DEGs were selected for qRT-PCR analysis. As a result, the transcript levels obtained by qRT-PCR showed similar expression trends to the corresponding genes in RNA-seq data, confirming the reliability of transcriptome analysis (**Figure 2**).

### 3.3 Genome-wide analysis of cold-responsive transcription factors

**Figure 3:**
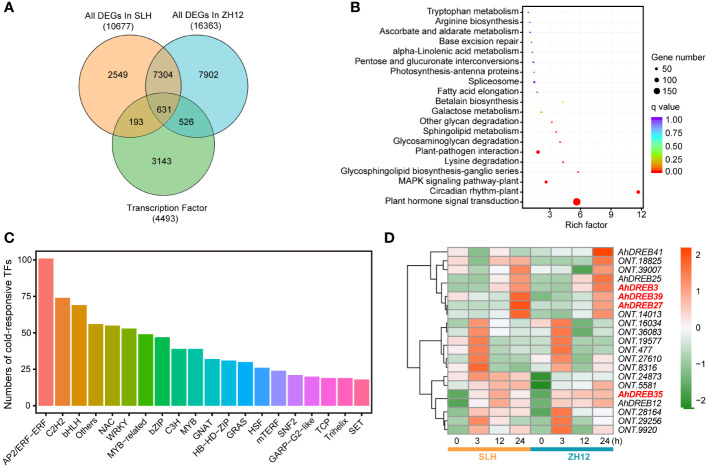
Analysis of cold responsive transcription factors in the transcriptome. **(A)** Venn diagram of cold responsive TFs in SLH and ZH12 peanut cultivars. KEGG enrichment **(B)** and family number **(C)** analysis of cold responsive TFs in this transcriptome. **(D)** Heatmap depicting expression pattern of *AhDREB* TFs. The red color in the box indicates high expression level, while green represents low.

Since transcription factors (TFs) played a crucial role in responding to cold stress, we performed genome-wide analysis of genes containing functional domains of TFs family in the two peanut cultivars. In total, 4,493 TFs were identified in our transcriptome data (**Supplementary Table S9**). The largest number of TF family was the bHLH member, followed by AP2/ethylene-responsive transcription (AP2/ERF) and MYB (**Supplementary Figure S2**). A total of 1,350 cold-responsive TFs were discovered in the two peanut cultivars (**Supplementary Table S10**). Among of them, 526 specific TFs had differential expression levels after cold treatment in ZH12, while only 193 specific TFs in SLH (**Figure 3A**). These 1,350 cold-responsive TFs were most significantly enriched in “plant hormone signal transduction” (ko04075) and “circadian rhythm-plant” (ko04712) KEGG pathways (**Figure 3B**). The AP2/ERF members accounted for the largest proportion of cold-responsive TFs (**Figure 3C**). CBFs/DREBs, belonging to AP2/ERF family, were extensively studied TFs that have important roles in plant cold acclimation (Kim et al., 2015; Tang et al., 2020). We identified 28 *AhDREBs* in the transcriptome (**Supplementary Table S11**), including *AhDREB3* (*Arahy.SE8WTS*), *AhDREB27* (*Arahy.KQL0FY*), *AhDREB35* (*Arahy.5KI8FH*), and *AhDREB39* (*Arahy.L5HMHT*). Most of them were significantly upregulated under cold stress in both SLH and ZH12 (**Figure 3D**). In addition, the 193 SLH-specific TFs were most enriched in “plant hormone signal transduction” KEGG pathway, with AP2/ERF members being the largest (**Supplementary Figure S3**). These results indicated that AP2/ERF play vital roles in signal transduction in adapting peanut to cold stress.

### 3.4 Analysis of alternative polyadenylation and alternative splicing events

**Figure 4:**
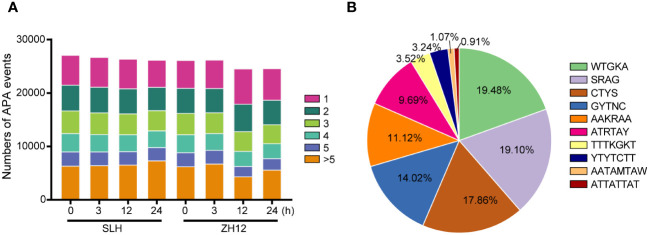
Analysis of alternative polyadenylation events. **(A)** The number of alternative polyadenylation (APA) events. **(B)** The top 10 APA motifs in the transcriptome.

As mentioned above, we identified 95,201 novel isoforms in our transcriptome (**Supplementary Table S5**). Most of these novel isoforms were probably derived from different forms of alternative splicing (AS) and extensions or truncations at 5′ or 3′ ends of mRNA. Polyadenylated (PolyA) tail, a long chain of adenine nucleotides, is added to the 3′ untranslated region (3′ UTR) of mRNA molecules for regulating their stability and translation (Jia et al., 2021). Due to presence of two or more poly A signals within the transcript, alternative polyadenylation (APA) events often occur to generate more isoforms and increase the diversity of transcriptome (Tu et al., 2020). We performed analysis of the characteristics of APA sites in the two peanut cultivars (**Figure 4A**; **Supplementary Table S12**). It was found that more than SLH (21,568) had more genes with APA sites than ZH12 (20,956) under normal condition (0 h). After cold treatment, the number of gene that possessed more than five APA sites was significantly increased in SLH, while other types of APA events were decreased in this cultivar. In contrast, the number of gene with one APA site was raised in ZH12, and the numbers of the remaining types of APA events were down. This result indicated that the cold stress promoted more APA sites that occurred in SLH (cold resistant) but that was inhibited in ZH12 (cold sensitive). In addition, the top 10 putative polyadenylation motifs were identified in the transcriptome data. The APA sites were most enriched in the motif WTGKA, accounting for 19.48% in the top 10 motifs (**Figure 4B**; **Supplementary Table S13**).

**Figure 5:**
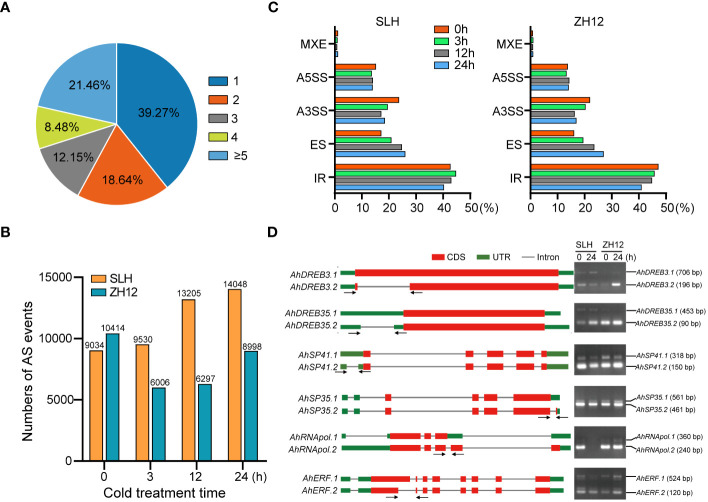
Analysis of alternative splicing events in the two peanut cultivars. **(A)** Statistics of genes with different number of alternative splicing (AS) isoforms. Summary of the numbers **(B)** and percentages **(C)** of different types of AS events during the cold stress treatment in the two peanut cultivars. **(D)** RT-PCR verification of AS isoforms in the transcriptome.

AS is an important posttranscriptional process of gene expression that affects the stability of transcripts and increases the diversity of proteins. A total of 23,673 (60.7%) genes were found to possess two or more alternative splicing variants in this transcriptome (**Figure 5A**; **Supplementary Table S14**). The number of AS events was significantly increased in SLH, while it was decreased in ZH12 under cold conditions (**Figure 5B**). This result indicated the differential impact of cold stress on AS events in the two cultivars. Moreover, five major types of AS events were discovered in the samples, including exon skipping (ES), intron retention (IR), mutually exclusive exon (MXE), alternative 5′ splice sites (A5SS), and alternative 3′ splice sites (A3SS). IR was the most abundant type of AS events and accounted for more than 40% of all AS events in SLH and ZH12 (**Figure 5C**; **Supplementary Table S15**). In both cultivars, after cold treatment, the proportion of A3SS and IR events decreased while ES increased compared to their normal controls (0 h). We performed RT-PCR analysis of six selected genes, including *DREBs* and splicing factors, to validate the cold-responsive alternative splicing events. The results showed that the alternative splicing patterns were consistent with their profiles revealed by ONT sequencing. As shown in **Figure 5D**, *AhDREB3* had two AS transcripts. The larger size of amplicon was *AhDREB3.1* (706 bp), which possessed an IR event in the coding region of the primary transcript, while the smaller one corresponded to *AhDREB3.2* (196 bp). Similar results were also observed in the other four genes (*AhDREB35*, *AhSP41*, *AhSP35*, and *AhRNApol*). On the other hand, *AhERF* underwent an A5SS event in the second exon of the primary mRNA, which resulted in two AS transcripts. The larger size of amplicon was *AhERF.1* (524 bp), and the smaller one corresponded to *AhERF.2* (120 bp). There was no positive signal in the second lane for *AhRNApol* gene, which was probably due to the fact that the transcript level of *AhRNApol* was substantially decreased after 24 h cold treatment in SLH.

### 3.5 Identification of differential alternative splicing genes in response to cold stress

**Figure 6:**
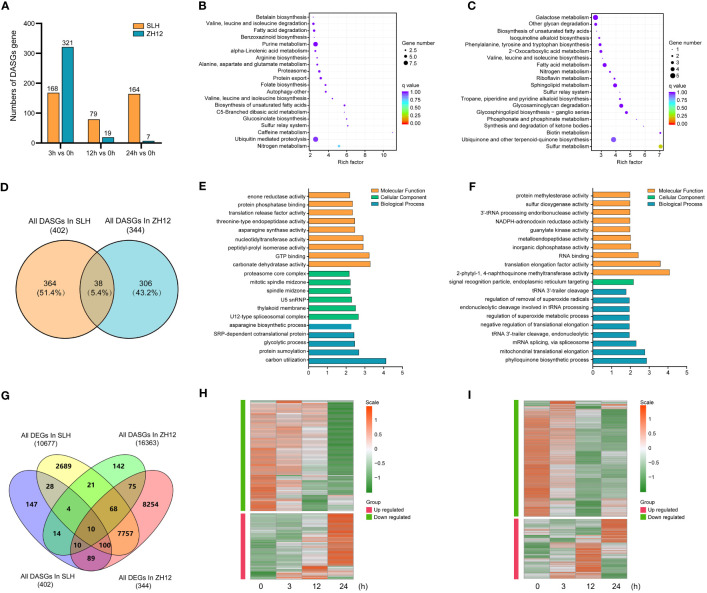
Comparative analysis of differential alternative splicing genes in response to cold stress in the two cultivars. **(A)** The numbers of differential alternative splicing genes (DASGs) during cold stress. KEGG enrichment analysis of DASGs in SLH **(B)** and ZH12 **(C)**. **(D)** Venn diagram of DASGs in the two cultivars. Gene Ontology enrichment analysis of DASGs in SLH **(E)** and ZH12 **(F)**. **(G)** Venn diagram of DASGs and DEGs. Heatmap shows the transcriptional expression pattern of DEGs with DAS events in SLH **(H)** and ZH12 **(I)**.

DAS events were detected between cold-treated samples (3 h, 12 h, and 24 h) and normal control (0 h). There was a total of 402 differential alternative splicing genes (DASGs) in the three comparison sets (24 h vs. 0 h, 12 h vs. 0 h, 3 h vs. 0 h) of SLH and 344 DASGs in ZH12, respectively (**Supplementary Table S16**). Unlike DEGs, the counts of gene with DAS events greatly decreased in ZH12 but slightly declined in SLH after 24 h of cold treatment (**Figure 6A**). To investigate the effect of cold-induced alternative splicing on cellular processes, we performed functional GO category and KEGG enrichment analysis of DASGs in the two cultivars. The DASGs in SLH had different enriched GO categories and KEGG pathways with those in ZH12 (**Figures 6B–F**). For instance, “nitrogen metabolism” and “ubiquitin mediated proteolysis” were the most enriched pathways in SLH, while “sulfur metabolism” and “ubiquinone and other terpenoid–quinone biosynthesis” were top enriched in ZH12. Next, a comparison was made between DASGs and DEGs to investigate how many DASGs were also changed their transcriptional levels under cold stress (**Figure 6G**). A total of 295 DASGs were differentially expressed in response to cold stress, while 427 DASGs did not have altered transcript levels. Moreover, we identified 142 DEGs with DAS events in SLH and 165 DEGs with DAS events in ZH12 (**Supplementary Tables S17**, **S18**). It has been shown that cold-induced DAS events often led to the formation of premature termination codons (PTCs) (Shen et al., 2016; Li et al., 2020a). Most of DEGs with DAS events were downregulated or degraded under cold stress in both of the two cultivars (**Figures 6H, I**).

### 3.6 Overexpression of *AhTPP* improves cold tolerance in transgenic yeast

**Figure 7:**
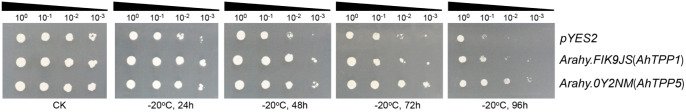
Overexpression of *AhTPPs* in yeast enhances tolerance to cold stress. The transgenic yeast cells harboring *AhTPPs* or pYES2 (empty vector control) were subjected to −20°C for different time, gradually diluted to different concentrations, and finally spotted onto SG-U solid plates. The yeast without cold treatment was used as control (CK).

In our previous study, we have developed a highly efficient yeast functional system to characterize potential cold tolerance genes in peanut (Wang et al., 2023). Based on our ONT transcriptome data, we selected two candidate genes (Arahy.FIK9JS and Arahy.0FY2NM) to verify their functions using the yeast platform. Both of the genes were significantly upregulated by low temperature in the two peanut cultivars and encoded putative trehalose-6-phosphate phosphatases (AhTPPs) (**Supplementary Table S6**). The two *AhTPPs* were transformed into yeast cells individually, and their cold tolerance were evaluated after low temperature treatment. As shown in **Figure 7**, the yeast transformants overexpressing *AhTPPs* exhibited better growth than the cells harboring pYES2 empty vector (control). Specifically, after cold treatment for 96 h, the control yeast cannot survive on the solid medium when the cultures were diluted to 10^−3^, whereas the transgenic yeast cells containing *AhTPP* were still able to grow under the same stressful conditions. These results indicated that overexpression of *AhTPP* improves cold tolerance in transgenic yeast.

## 4 Discussion

As most peanut cultivars are thermophilic, the ability to tolerate low temperature is a major bottleneck restricting their cultivation and production. Understanding of the molecular mechanism of cold tolerance will provide a basis to develop cold-resistant cultivars, which is required for expanding their cultivated regions to high altitude and/or high-latitude chilling areas. Transcriptional regulation of gene expression and adjustment of metabolism are important ways for crops to deal with cold stress. In our previous study, integrated SGS-based transcriptomic and metabolomic analysis revealed a set of important cold-responsive genes and key metabolism pathways contributing to cold tolerance in peanut (Wang et al., 2021). However, the SGS-based RNA-seq technology is often unable to assemble the entire transcripts and to recognize transcript isoforms of homologous gene, superfamily gene, and allele expression; it will be hard for us to further elucidate cold responsive processes in peanut. In the present study, we investigated gene regulation under cold stress in the two peanut varieties by using ONT sequencing technology, which could obtain high-quality full-length transcript sequences without needing assemble the reads. Totally, 8,949 novel gene loci (23.2% of total loci) and 95,291 new/novel isoforms (76.06% of total transcripts) were detected, indicating that the ONT sequencing was a useful platform for discovering novel genes and transcripts in the available peanut genome.

In addition, we were able to identify DEG and AS events and to study how they were affected by cold stress. A total of 2,742 DEGs were found to be responsive to cold stress in SLH specifically. These genes might play positive and/or negative roles in regulating cold resistance in peanut. For example, the expression of *Arahy.51NNVX*, encoding a homolog of transcriptional adapter ADA2, was specifically downregulated by low temperature in SLH. A disruption of *ADA2b* in *Arabidopsis* improved freezing tolerance compared with wild-type plants under non-acclimated condition, indicating that ADA2b acted as a repressor in response to cold stress (Vlachonasios et al., 2003). APA is a widespread mechanism that regulates various developmental processes and multiple abiotic stress responses in plant (Chakrabarti et al., 2020; Zhou and Li, 2023). Approximately 70% of *Arabidopsis* genes and 48% of rice genes have two and more selective poly(A) sites (Wu et al., 2011; Fu et al., 2016). Similarly, more than 48% of peanut genes underwent APA events in our transcriptome. The number of genes with APA events in SLH was larger than that in ZH12 under both normal and cold stress conditions. Specifically, the number of genes that possessed more than five APA sites was increased in SLH during cold stress but was decreased in ZH12. This led to more increment of transcriptome diversity and complexity in SLH and probably contributed to its adaptation to cold stressful condition. Previous reports also had showed that AS events were regulated by multiple environmental stress and were largely associated with cold stress resistance in plants (Li et al., 2020b; John et al., 2021; Yang et al., 2021). In agreement with other plant species, the predominant AS events were IR in peanut. The number of IR events was decreased after cold treatment, while ES was significantly increased in both of the peanut cultivars; similar results were also found in quinoa and other plants (Zheng et al., 2022a, b). In addition, our transcriptome data showed that more DEGs were identified in cold-sensitive cultivar (ZH12) than in cold-tolerant cultivar (SLH), while more AS events were found in SLH compared to ZH12. These results suggested that the two peanut cultivars may undergo differential mechanisms to respond to cold stress. It has been reported that the nuclear cap-binding complex (CBC) plays a crucial role in abiotic stress response (Daszkowska-Golec, 2018). The two subunits of *Arabidopsis* CBC (AtCBP20 and AtCBP80) could be a platform for interacting with other splicing factors and thus involved in alternative splicing of some genes under stressful conditions. Overexpression of a CBC subunit gene from *D. catenatum* (*DcCBP20*) in *Arabidopsis* led to the generation of more AS isoforms of cold-induced genes compared with wild-type plants (Zheng et al., 2022b). Four genes (*Arahy.VD4ZCS*, *Arahy.RWFH5R*, *Arahy.ZCX2T9*, and *Arahy.UE89WV*) were annotated as CBC subunits in our transcriptome. Among them, *Arahy.RWFH5R* was found to be downregulated under cold stress specifically in ZH12, which might affect the profiles of AS and lead to a decrease in the number of AS events in ZH12. However, the molecular mechanism of CBC in regulating peanut cold response warrant further study.

DAS produced different types of isoforms under cold stress compared with control. Splicing factors and their associated components play important roles in alternative splicing during the post-transcription processing (Zhao et al., 2021). We found 292 genes related to RNA splicing and processing in the transcriptome; most of these genes were annotated with KEGG pathway “spliceosome” (ko03040). Among them, 101 genes altered their expression level, while only eight genes had DAS events in response to cold stress. For instance, serine/arginine-rich (SR) splicing factors has been reported to be involved in abiotic stress response by regulating AS processes (Tanabe et al., 2007; Li et al., 2021). Disruption of *Arabidopsis SR* genes affects the splicing patterns of their own pre-mRNA and downstream target genes (Palusa et al., 2007; Yan et al., 2017). We detected 27 *SR* splicing factors in our transcriptome. Among of them, eight *SR* genes changes their expression level, while three *SR* had DAS events in response to cold stress. Notably, one SR (*arahy.9ADW36*) had both differential expression level and AS event. These results suggested that the changes in the transcript variants of *SP* splicing factors would affect both the transcription patterns and AS events of downstream target genes under low temperature conditions.

Trehalose is a non-reducing disaccharide that is commonly found in bacteria, yeast, and plants (Lunn et al., 2014). Plant trehalose biosynthesis is a conserved two-step pathway involving trehalose 6-phosphate synthases (TPS) and TPP enzymes, which are involved in protecting the structure of cells and bioactive molecules under stressful conditions, such as low temperature, drought, and high salinity (Kerbler et al., 2023). In this study, two peanut *AhTPPs* (*Arahy.FIK9JS* and *Arahy.0FY2NM*) were identified as cold tolerance candidate genes by ONT transcriptome sequencing. Introducing either of them in yeast cells could confer enhanced tolerance to cold stress. Previous report had shown that overexpression of *OsTPP1* in rice led to increased cold tolerance (Ge et al., 2008). These results indicated that combining ONT transcriptome and yeast functional screening system was an effective approach for discovering potential cold tolerance genes in plants. Moreover, the two *AhTPPs* and the other identified candidate genes would be used as valuable gene resources for genetic improvement of abiotic stress in peanut and other crops.

## 5 Conclusion

In this study, we analyzed the full-length transcriptome of two peanut cultivars using ONT sequencing platform and obtained three main findings. First, a total of 8,949 novel gene loci and 95,291 new/novel isoforms were discovered by comparing with available reference genome and/or transcriptome. Second, the increased APA and AS events in SLH during cold stress led to rising transcriptome diversity and complexity, which probably contributed to its stronger cold tolerance in comparison to ZH12. Finally, the identified 28 cold-induced AhDREBs and eight SR splicing factors with DAS events might play vital roles in peanut cold response. This research enriched the full-length transcriptome and genomic data of peanut. The findings of the candidate cold responsive genes will contribute to breeding of peanut variety with increased abiotic stress tolerance in future.

## Data availability statement

The datasets presented in this study can be found in online repositories. The names of the repository/repositories and accession number(s) can be found in the article/**Supplementary Material**.

## Author contributions

XW: Funding acquisition, Investigation, Writing – original draft, Writing – review & editing. YLiu: Investigation, Validation, Writing – original draft. LO: Investigation, Validation, Writing – original draft. RY: Investigation, Validation, Writing – original draft. TY: Investigation, Validation, Writing – original draft. LY: Data curation, Writing – original draft. YC: Data curation, Writing – original draft. DH: Data curation, Writing – original draft. XZ: Data curation, Writing – original draft, Funding acquisition. ZW: Data curation, Writing – original draft, Software. YK: Data curation, Writing – original draft, Formal analysis. WQ: Writing – original draft, Data curation, Methodology. HJ: Conceptualization, Supervision, Writing – original draft, Writing – review & editing, Resources. YLei: Funding acquisition, Supervision, Writing – review & editing, Writing – original draft, Conceptualization. BL: Funding acquisition, Writing – original draft, Supervision, Writing – review & editing.
