# Supplementary material for: Full-length transcriptome sequencing provides insights into alternative splicing under cold stress in peanut
Source: Front Plant Sci. 2024 Mar 7;15:1362277. doi: 10.3389/fpls.2024.1362277 (PMC10954824; doi:10.3389/fpls.2024.1362277)
Supplement: Supplementary file 1 [file DataSheet_1.pdf]

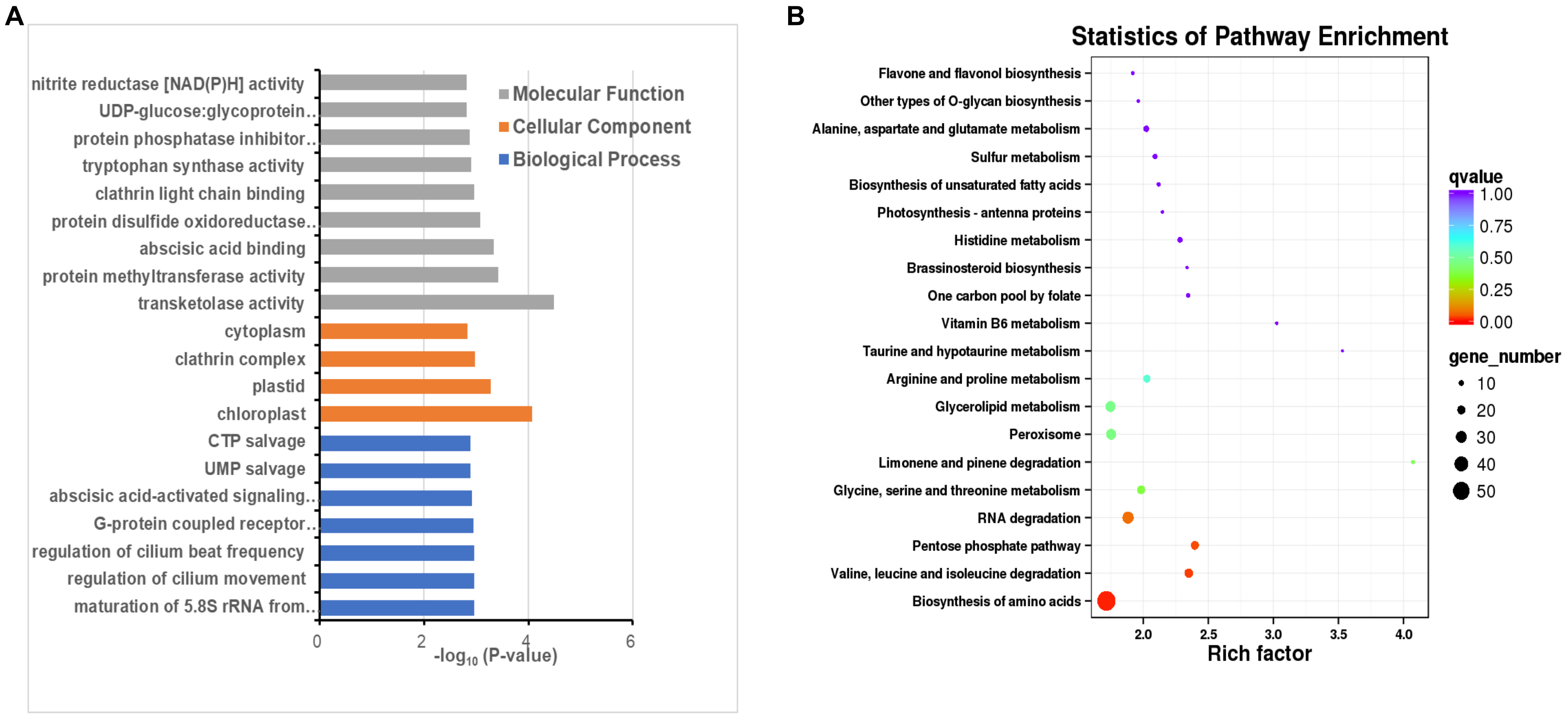

Figure S1. Gene ontology (A) and KEGG (B) enrichment analysis of differentially expressed genes found specifically in SLH peanut cultivars.

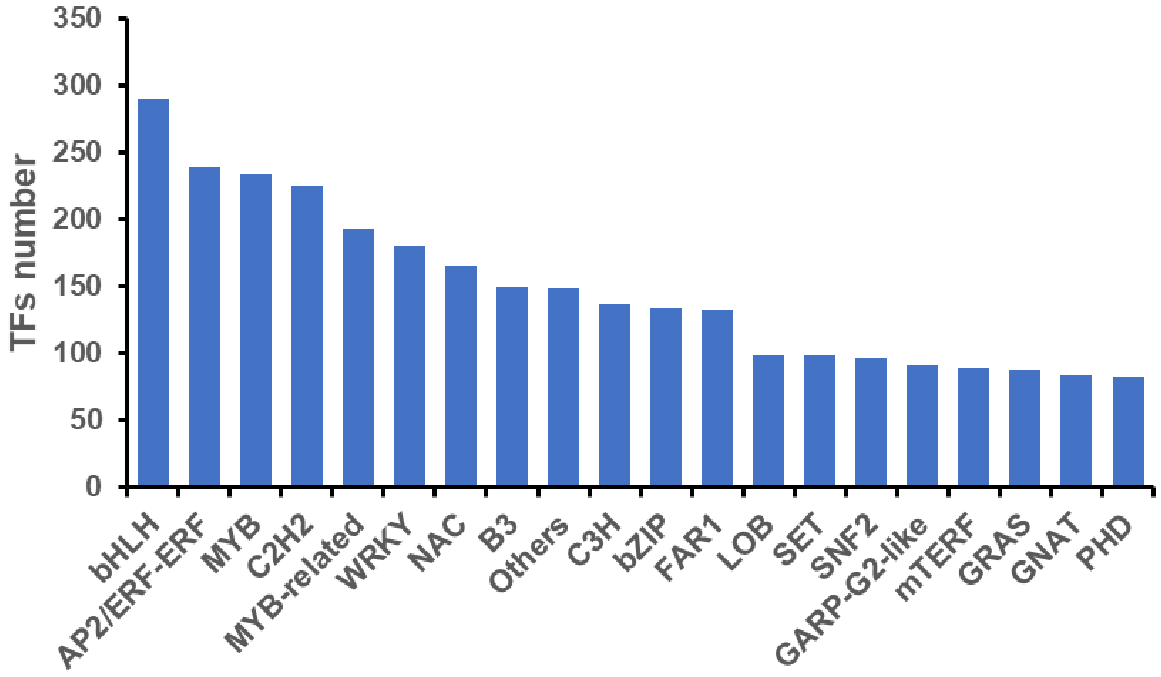

Figure S2. Statistics of transcript factor families in this transcriptome.

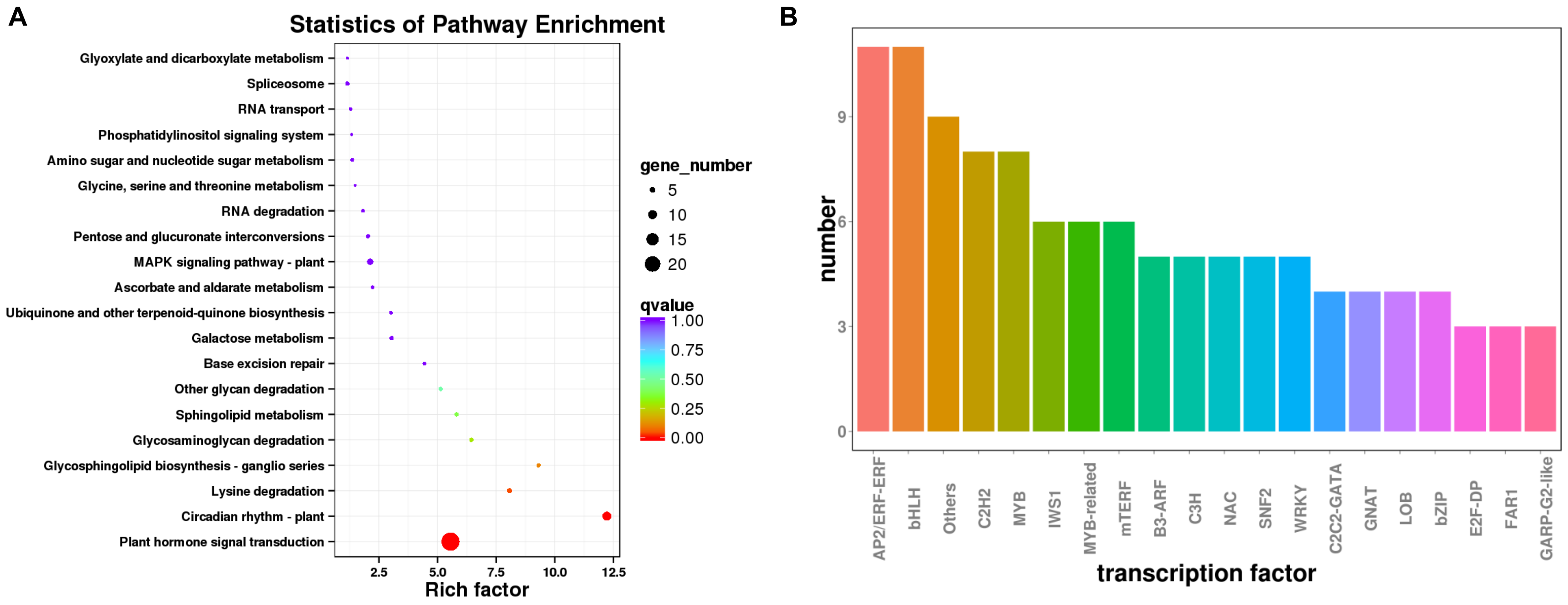

Figure S3. KEGG enrichment analysis (A) and family number (B) of cold responsive transcription factors specifically in SLH peanut cultivars.
